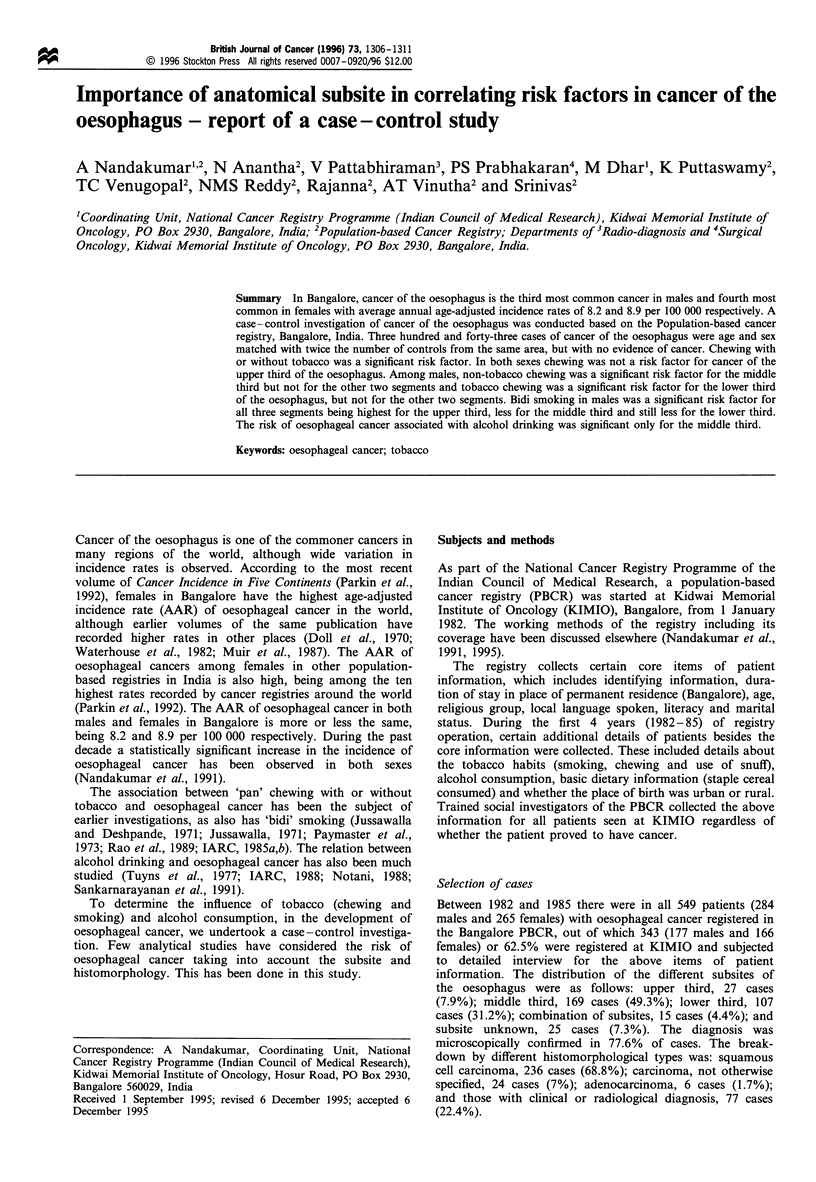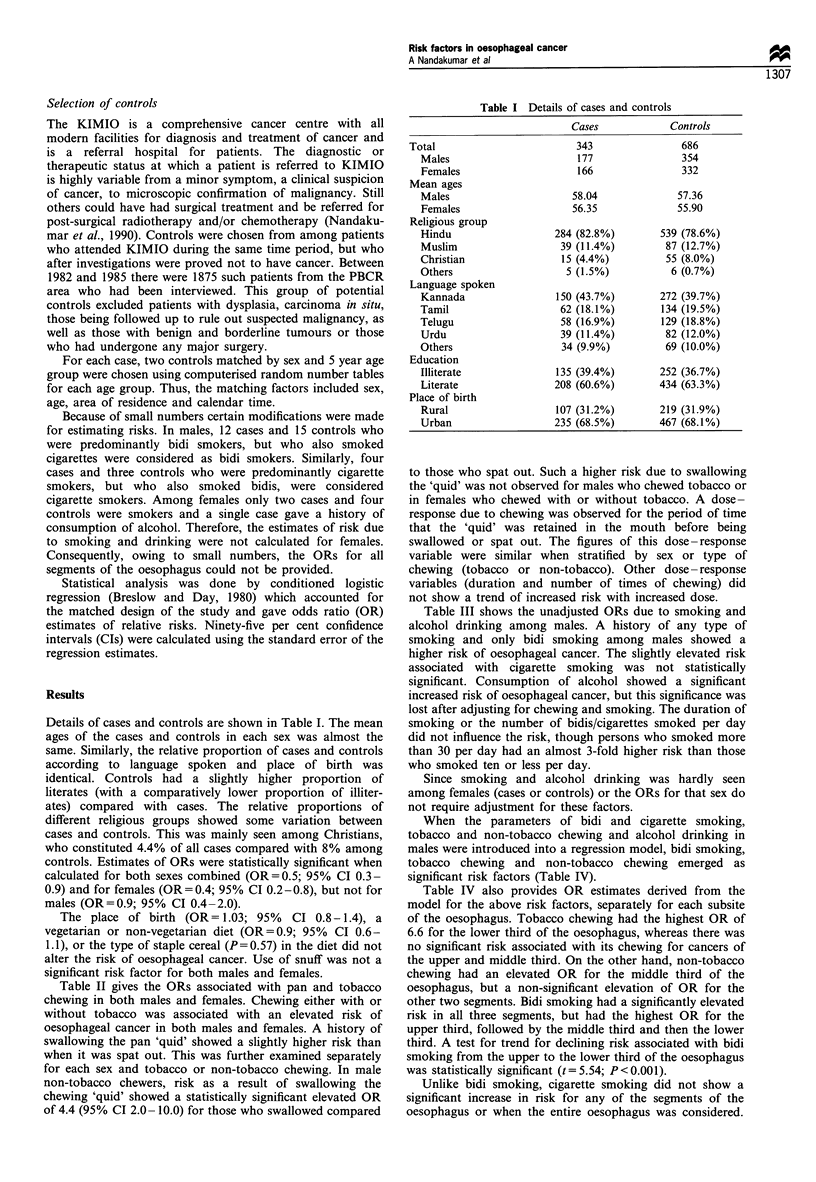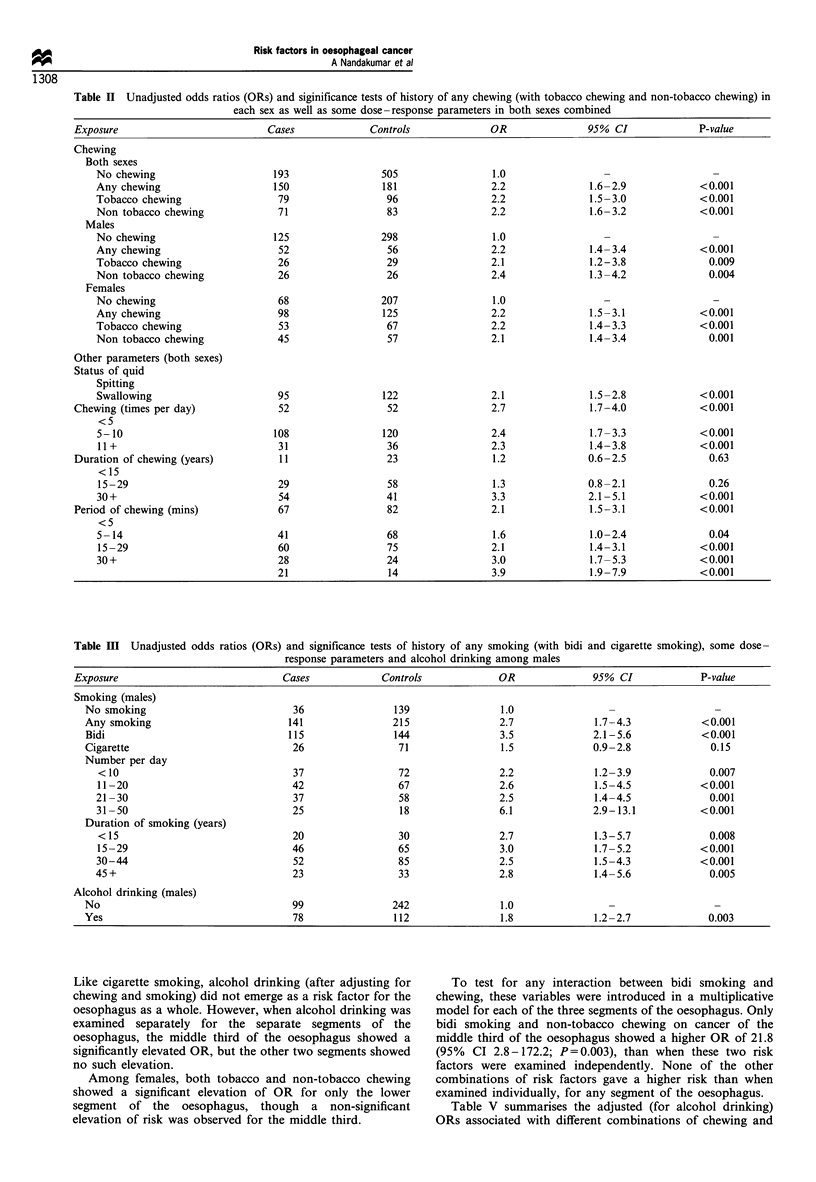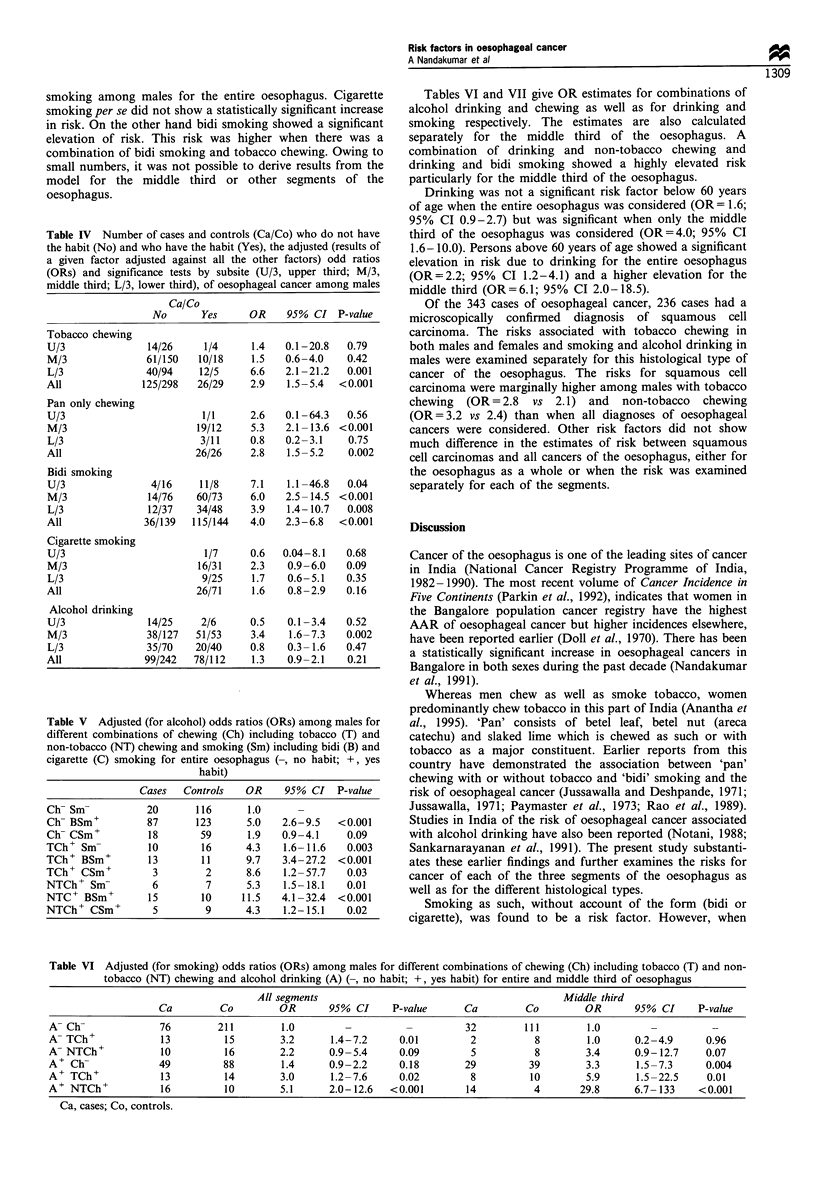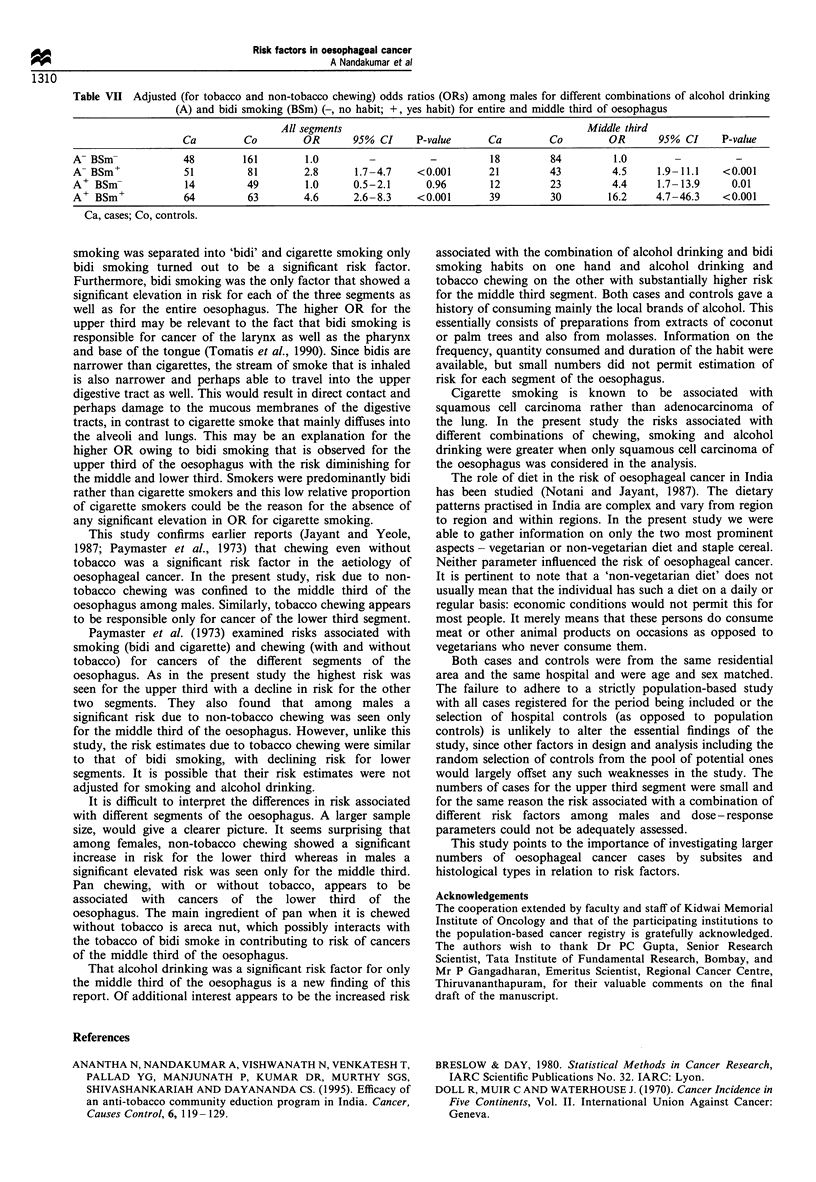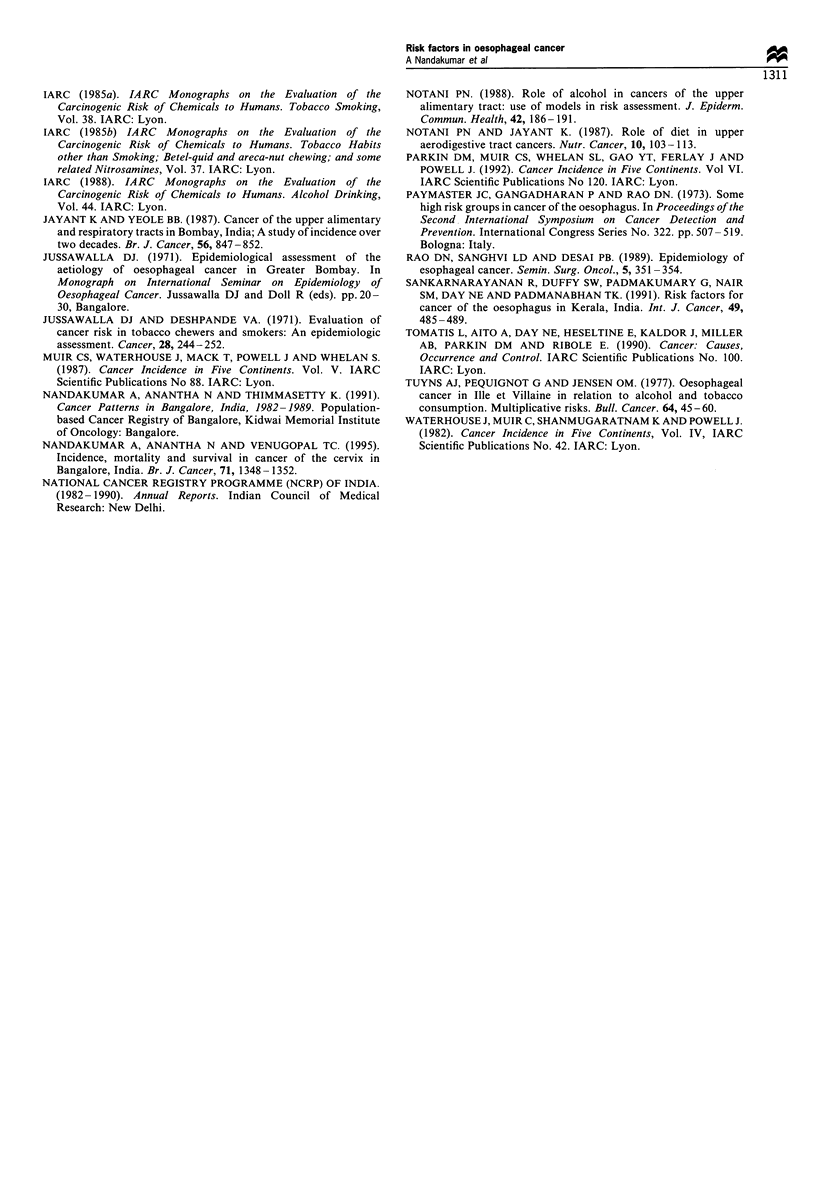# Importance of anatomical subsite in correlating risk factors in cancer of the oesophagus--report of a case--control study.

**DOI:** 10.1038/bjc.1996.249

**Published:** 1996-05

**Authors:** A. Nandakumar, N. Anantha, V. Pattabhiraman, P. S. Prabhakaran, M. Dhar, K. Puttaswamy, T. C. Venugopal, N. M. Reddy, A. T. Vinutha

**Affiliations:** National Cancer Registry Programme (Indian Council of Medical Research), Kidwai Memorial Institute of Oncology, Bangalore, India.

## Abstract

In Bangalore, cancer of the oesophagus is the third most common cancer in males and fourth most common in females with average annual age-adjusted incidence rates of 8.2 and 8.9 per 100,000 respectively. A case-control investigation of cancer of the oesophagus was conducted based on the Population-based cancer registry, Bangalore, India. Three hundred and forty-three cases of cancer of the oesophagus were age and sex matched with twice the number of controls from the same area, but with no evidence of cancer. Chewing with or without tobacco was a significant risk factor. In both sexes chewing was not a risk factor for cancer of the upper third of the oesophagus. Among males, non-tobacco chewing was a significant risk factor for the middle third but not for the other two segments and tobacco chewing was a significant risk factor for the lower third of the oesophagus, but not for the other two segments. Bidi smoking in males was a significant risk factor for all three segments being highest for the upper third, less for the middle third and still less for the lower third. The risk of oesophageal cancer associated with alcohol drinking was significant only for the middle third.


British Journal of Cancer (1996) 73, 1306-1311
© 1996 Stockton Press All rights reserved 0007-0920/96 $12.00

### Importance of anatomical subsite in correlating risk factors in cancer of the oesophagus – report of a case–control study

A Nandakumar[1,2], N Anantha[2], V Pattabhiraman[3], PS Prabhakaran[4], M Dhar[1], K Puttaswamy[2], TC Venugopal[2], NMS Reddy[2], Rajanna[2], AT Vinutha[2] and Srinivas[2]

[1]*Coordinating Unit, National Cancer Registry Programme (Indian Council of Medical Research), Kidwai Memorial Institute of Oncology, PO Box 2930, Bangalore, India;* [2]*Population-based Cancer Registry; Departments of* [3]*Radio-diagnosis and* [4]*Surgical Oncology, Kidwai Memorial Institute of Oncology, PO Box 2930, Bangalore, India.*

**Summary** In Bangalore, cancer of the oesophagus is the third most common cancer in males and fourth most common in females with average annual age-adjusted incidence rates of 8.2 and 8.9 per 100 000 respectively. A case–control investigation of cancer of the oesophagus was conducted based on the Population-based cancer registry, Bangalore, India. Three hundred and forty-three cases of cancer of the oesophagus were age and sex matched with twice the number of controls from the same area, but with no evidence of cancer. Chewing with or without tobacco was a significant risk factor. In both sexes chewing was not a risk factor for cancer of the upper third of the oesophagus. Among males, non-tobacco chewing was a significant risk factor for the middle third but not for the other two segments and tobacco chewing was a significant risk factor for the lower third of the oesophagus, but not for the other two segments. Bidi smoking in males was a significant risk factor for all three segments being highest for the upper third, less for the middle third and still less for the lower third. The risk of oesophageal cancer associated with alcohol drinking was significant only for the middle third.

**Keywords:** oesophageal cancer; tobacco

Cancer of the oesophagus is one of the commoner cancers in many regions of the world, although wide variation in incidence rates is observed. According to the most recent volume of *Cancer Incidence in Five Continents* (Parkin *et al.*, 1992), females in Bangalore have the highest age-adjusted incidence rate (AAR) of oesophageal cancer in the world, although earlier volumes of the same publication have recorded higher rates in other places (Doll *et al.*, 1970; Waterhouse *et al.*, 1982; Muir *et al.*, 1987). The AAR of oesophageal cancers among females in other population-based registries in India is also high, being among the ten highest rates recorded by cancer registries around the world (Parkin *et al.*, 1992). The AAR of oesophageal cancer in both males and females in Bangalore is more or less the same, being 8.2 and 8.9 per 100 000 respectively. During the past decade a statistically significant increase in the incidence of oesophageal cancer has been observed in both sexes (Nandakumar *et al.*, 1991).

The association between 'pan' chewing with or without tobacco and oesophageal cancer has been the subject of earlier investigations, as also has 'bidi' smoking (Jussawalla and Deshpande, 1971; Jussawalla, 1971; Paymaster *et al.*, 1973; Rao *et al.*, 1989; IARC, 1985*a*,*b*). The relation between alcohol drinking and oesophageal cancer has also been much studied (Tuyns *et al.*, 1977; IARC, 1988; Notani, 1988; Sankarnarayanan *et al.*, 1991).

To determine the influence of tobacco (chewing and smoking) and alcohol consumption, in the development of oesophageal cancer, we undertook a case–control investigation. Few analytical studies have considered the risk of oesophageal cancer taking into account the subsite and histomorphology. This has been done in this study.

Correspondence: A Nandakumar, Coordinating Unit, National Cancer Registry Programme (Indian Council of Medical Research), Kidwai Memorial Institute of Oncology, Hosur Road, PO Box 2930, Bangalore 560029, India
Received 1 September 1995; revised 6 December 1995; accepted 6 December 1995

### Subjects and methods

As part of the National Cancer Registry Programme of the Indian Council of Medical Research, a population-based cancer registry (PBCR) was started at Kidwai Memorial Institute of Oncology (KIMIO), Bangalore, from 1 January 1982. The working methods of the registry including its coverage have been discussed elsewhere (Nandakumar *et al.*, 1991, 1995).

The registry collects certain core items of patient information, which includes identifying information, duration of stay in place of permanent residence (Bangalore), age, religious group, local language spoken, literacy and marital status. During the first 4 years (1982–85) of registry operation, certain additional details of patients besides the core information were collected. These included details about the tobacco habits (smoking, chewing and use of snuff), alcohol consumption, basic dietary information (staple cereal consumed) and whether the place of birth was urban or rural. Trained social investigators of the PBCR collected the above information for all patients seen at KIMIO regardless of whether the patient proved to have cancer.

### *Selection of cases*

Between 1982 and 1985 there were in all 549 patients (284 males and 265 females) with oesophageal cancer registered in the Bangalore PBCR, out of which 343 (177 males and 166 females) or 62.5% were registered at KIMIO and subjected to detailed interview for the above items of patient information. The distribution of the different subsites of the oesophagus were as follows: upper third, 27 cases (7.9%); middle third, 169 cases (49.3%); lower third, 107 cases (31.2%); combination of subsites, 15 cases (4.4%); and subsite unknown, 25 cases (7.3%). The diagnosis was microscopically confirmed in 77.6% of cases. The breakdown by different histomorphological types was: squamous cell carcinoma, 236 cases (68.8%); carcinoma, not otherwise specified, 24 cases (7%); adenocarcinoma, 6 cases (1.7%); and those with clinical or radiological diagnosis, 77 cases (22.4%).

*Selection of controls*

The KIMIO is a comprehensive cancer centre with all modern facilities for diagnosis and treatment of cancer and is a referral hospital for patients. The diagnostic or therapeutic status at which a patient is referred to KIMIO is highly variable from a minor symptom, a clinical suspicion of cancer, to microscopic confirmation of malignancy. Still others could have had surgical treatment and be referred for post-surgical radiotherapy and/or chemotherapy (Nandakumar *et al.*, 1990). Controls were chosen from among patients who attended KIMIO during the same time period, but who after investigations were proved not to have cancer. Between 1982 and 1985 there were 1875 such patients from the PBCR area who had been interviewed. This group of potential controls excluded patients with dysplasia, carcinoma *in situ*, those being followed up to rule out suspected malignancy, as well as those with benign and borderline tumours or those who had undergone any major surgery.

For each case, two controls matched by sex and 5 year age group were chosen using computerised random number tables for each age group. Thus, the matching factors included sex, age, area of residence and calendar time.

Because of small numbers certain modifications were made for estimating risks. In males, 12 cases and 15 controls who were predominantly bidi smokers, but who also smoked cigarettes were considered as bidi smokers. Similarly, four cases and three controls who were predominantly cigarette smokers, but who also smoked bidis, were considered cigarette smokers. Among females only two cases and four controls were smokers and a single case gave a history of consumption of alcohol. Therefore, the estimates of risk due to smoking and drinking were not calculated for females. Consequently, owing to small numbers, the ORs for all segments of the oesophagus could not be provided.

Statistical analysis was done by conditioned logistic regression (Breslow and Day, 1980) which accounted for the matched design of the study and gave odds ratio (OR) estimates of relative risks. Ninety-five per cent confidence intervals (CIs) were calculated using the standard error of the regression estimates.

### Results

Details of cases and controls are shown in Table I. The mean ages of the cases and controls in each sex was almost the same. Similarly, the relative proportion of cases and controls according to language spoken and place of birth was identical. Controls had a slightly higher proportion of literates (with a comparatively lower proportion of illiterates) compared with cases. The relative proportions of different religious groups showed some variation between cases and controls. This was mainly seen among Christians, who constituted 4.4% of all cases compared with 8% among controls. Estimates of ORs were statistically significant when calculated for both sexes combined (OR = 0.5; 95% CI 0.3–0.9) and for females (OR = 0.4; 95% CI 0.2–0.8), but not for males (OR = 0.9; 95% CI 0.4–2.0).

The place of birth (OR = 1.03; 95% CI 0.8–1.4), a vegetarian or non-vegetarian diet (OR = 0.9; 95% CI 0.6–1.1), or the type of staple cereal ($P = 0.57$) in the diet did not alter the risk of oesophageal cancer. Use of snuff was not a significant risk factor for both males and females.

Table II gives the ORs associated with pan and tobacco chewing in both males and females. Chewing either with or without tobacco was associated with an elevated risk of oesophageal cancer in both males and females. A history of swallowing the pan 'quid' showed a slightly higher risk than when it was spat out. This was further examined separately for each sex and tobacco or non-tobacco chewing. In male non-tobacco chewers, risk as a result of swallowing the chewing 'quid' showed a statistically significant elevated OR of 4.4 (95% CI 2.0–10.0) for those who swallowed compared to those who spat out. Such a higher risk due to swallowing the 'quid' was not observed for males who chewed tobacco or in females who chewed with or without tobacco. A dose–response due to chewing was observed for the period of time that the 'quid' was retained in the mouth before being swallowed or spat out. The figures of this dose–response variable were similar when stratified by sex or type of chewing (tobacco or non-tobacco). Other dose–response variables (duration and number of times of chewing) did not show a trend of increased risk with increased dose.

**Table I** Details of cases and controls

| | *Cases* | *Controls* |
|---|---|---|
| Total | 343 | 686 |
| Males | 177 | 354 |
| Females | 166 | 332 |
| Mean ages | | |
| Males | 58.04 | 57.36 |
| Females | 56.35 | 55.90 |
| Religious group | | |
| Hindu | 284 (82.8%) | 539 (78.6%) |
| Muslim | 39 (11.4%) | 87 (12.7%) |
| Christian | 15 (4.4%) | 55 (8.0%) |
| Others | 5 (1.5%) | 6 (0.7%) |
| Language spoken | | |
| Kannada | 150 (43.7%) | 272 (39.7%) |
| Tamil | 62 (18.1%) | 134 (19.5%) |
| Telugu | 58 (16.9%) | 129 (18.8%) |
| Urdu | 39 (11.4%) | 82 (12.0%) |
| Others | 34 (9.9%) | 69 (10.0%) |
| Education | | |
| Illiterate | 135 (39.4%) | 252 (36.7%) |
| Literate | 208 (60.6%) | 434 (63.3%) |
| Place of birth | | |
| Rural | 107 (31.2%) | 219 (31.9%) |
| Urban | 235 (68.5%) | 467 (68.1%) |

Table III shows the unadjusted ORs due to smoking and alcohol drinking among males. A history of any type of smoking and only bidi smoking among males showed a higher risk of oesophageal cancer. The slightly elevated risk associated with cigarette smoking was not statistically significant. Consumption of alcohol showed a significant increased risk of oesophageal cancer, but this significance was lost after adjusting for chewing and smoking. The duration of smoking or the number of bidis/cigarettes smoked per day did not influence the risk, though persons who smoked more than 30 per day had an almost 3-fold higher risk than those who smoked ten or less per day.

Since smoking and alcohol drinking was hardly seen among females (cases or controls) or the ORs for that sex do not require adjustment for these factors.

When the parameters of bidi and cigarette smoking, tobacco and non-tobacco chewing and alcohol drinking in males were introduced into a regression model, bidi smoking, tobacco chewing and non-tobacco chewing emerged as significant risk factors (Table IV).

Table IV also provides OR estimates derived from the model for the above risk factors, separately for each subsite of the oesophagus. Tobacco chewing had the highest OR of 6.6 for the lower third of the oesophagus, whereas there was no significant risk associated with its chewing for cancers of the upper and middle third. On the other hand, non-tobacco chewing had an elevated OR for the middle third of the oesophagus, but a non-significant elevation of OR for the other two segments. Bidi smoking had a significantly elevated risk in all three segments, but had the highest OR for the upper third, followed by the middle third and then the lower third. A test for trend for declining risk associated with bidi smoking from the upper to the lower third of the oesophagus was statistically significant ($t = 5.54$; $P < 0.001$).

Unlike bidi smoking, cigarette smoking did not show a significant increase in risk for any of the segments of the oesophagus or when the entire oesophagus was considered.

**Table II** Unadjusted odds ratios (ORs) and siginificance tests of history of any chewing (with tobacco chewing and non-tobacco chewing) in each sex as well as some dose–response parameters in both sexes combined

| *Exposure* | *Cases* | *Controls* | *OR* | *95% CI* | P-*value* |
|---|---|---|---|---|---|
| Chewing | | | | | |
| Both sexes | | | | | |
| No chewing | 193 | 505 | 1.0 | – | – |
| Any chewing | 150 | 181 | 2.2 | 1.6–2.9 | <0.001 |
| Tobacco chewing | 79 | 96 | 2.2 | 1.5–3.0 | <0.001 |
| Non tobacco chewing | 71 | 83 | 2.2 | 1.6–3.2 | <0.001 |
| Males | | | | | |
| No chewing | 125 | 298 | 1.0 | – | – |
| Any chewing | 52 | 56 | 2.2 | 1.4–3.4 | <0.001 |
| Tobacco chewing | 26 | 29 | 2.1 | 1.2–3.8 | 0.009 |
| Non tobacco chewing | 26 | 26 | 2.4 | 1.3–4.2 | 0.004 |
| Females | | | | | |
| No chewing | 68 | 207 | 1.0 | – | – |
| Any chewing | 98 | 125 | 2.2 | 1.5–3.1 | <0.001 |
| Tobacco chewing | 53 | 67 | 2.2 | 1.4–3.3 | <0.001 |
| Non tobacco chewing | 45 | 57 | 2.1 | 1.4–3.4 | 0.001 |
| Other parameters (both sexes) | | | | | |
| Status of quid | | | | | |
| Spitting | | | | | |
| Swallowing | 95 | 122 | 2.1 | 1.5–2.8 | <0.001 |
| Chewing (times per day) | 52 | 52 | 2.7 | 1.7–4.0 | <0.001 |
| <5 | | | | | |
| 5–10 | 108 | 120 | 2.4 | 1.7–3.3 | <0.001 |
| 11+ | 31 | 36 | 2.3 | 1.4–3.8 | <0.001 |
| Duration of chewing (years) | 11 | 23 | 1.2 | 0.6–2.5 | 0.63 |
| <15 | | | | | |
| 15–29 | 29 | 58 | 1.3 | 0.8–2.1 | 0.26 |
| 30+ | 54 | 41 | 3.3 | 2.1–5.1 | <0.001 |
| Period of chewing (mins) | 67 | 82 | 2.1 | 1.5–3.1 | <0.001 |
| <5 | | | | | |
| 5–14 | 41 | 68 | 1.6 | 1.0–2.4 | 0.04 |
| 15–29 | 60 | 75 | 2.1 | 1.4–3.1 | <0.001 |
| 30+ | 28 | 24 | 3.0 | 1.7–5.3 | <0.001 |
| | 21 | 14 | 3.9 | 1.9–7.9 | <0.001 |

**Table III** Unadjusted odds ratios (ORs) and significance tests of history of any smoking (with bidi and cigarette smoking), some dose–response parameters and alcohol drinking among males

| *Exposure* | *Cases* | *Controls* | *OR* | *95% CI* | P-*value* |
|---|---|---|---|---|---|
| Smoking (males) | | | | | |
| No smoking | 36 | 139 | 1.0 | – | – |
| Any smoking | 141 | 215 | 2.7 | 1.7–4.3 | <0.001 |
| Bidi | 115 | 144 | 3.5 | 2.1–5.6 | <0.001 |
| Cigarette | 26 | 71 | 1.5 | 0.9–2.8 | 0.15 |
| Number per day | | | | | |
| <10 | 37 | 72 | 2.2 | 1.2–3.9 | 0.007 |
| 11–20 | 42 | 67 | 2.6 | 1.5–4.5 | <0.001 |
| 21–30 | 37 | 58 | 2.5 | 1.4–4.5 | 0.001 |
| 31–50 | 25 | 18 | 6.1 | 2.9–13.1 | <0.001 |
| Duration of smoking (years) | | | | | |
| <15 | 20 | 30 | 2.7 | 1.3–5.7 | 0.008 |
| 15–29 | 46 | 65 | 3.0 | 1.7–5.2 | <0.001 |
| 30–44 | 52 | 85 | 2.5 | 1.5–4.3 | <0.001 |
| 45+ | 23 | 33 | 2.8 | 1.4–5.6 | 0.005 |
| Alcohol drinking (males) | | | | | |
| No | 99 | 242 | 1.0 | – | – |
| Yes | 78 | 112 | 1.8 | 1.2–2.7 | 0.003 |

Like cigarette smoking, alcohol drinking (after adjusting for chewing and smoking) did not emerge as a risk factor for the oesophagus as a whole. However, when alcohol drinking was examined separately for the separate segments of the oesophagus, the middle third of the oesophagus showed a significantly elevated OR, but the other two segments showed no such elevation.

Among females, both tobacco and non-tobacco chewing showed a significant elevation of OR for only the lower segment of the oesophagus, though a non-significant elevation of risk was observed for the middle third.

To test for any interaction between bidi smoking and chewing, these variables were introduced in a multiplicative model for each of the three segments of the oesophagus. Only bidi smoking and non-tobacco chewing on cancer of the middle third of the oesophagus showed a higher OR of 21.8 (95% CI 2.8–172.2; $P=0.003$), than when these two risk factors were examined independently. None of the other combinations of risk factors gave a higher risk than when examined individually, for any segment of the oesophagus.

Table V summarises the adjusted (for alcohol drinking) ORs associated with different combinations of chewing and smoking among males for the entire oesophagus. Cigarette smoking *per se* did not show a statistically significant increase in risk. On the other hand bidi smoking showed a significant elevation of risk. This risk was higher when there was a combination of bidi smoking and tobacco chewing. Owing to small numbers, it was not possible to derive results from the model for the middle third or other segments of the oesophagus.

**Table IV** Number of cases and controls (Ca/Co) who do not have the habit (No) and who have the habit (Yes), the adjusted (results of a given factor adjusted against all the other factors) odd ratios (ORs) and significance tests by subsite (U/3, upper third; M/3, middle third; L/3, lower third), of oesophageal cancer among males

| | *Ca/Co* No | *Ca/Co* Yes | *OR* | *95% CI* | P-*value* |
|---|---|---|---|---|---|
| Tobacco chewing | | | | | |
| U/3 | 14/26 | 1/4 | 1.4 | 0.1–20.8 | 0.79 |
| M/3 | 61/150 | 10/18 | 1.5 | 0.6–4.0 | 0.42 |
| L/3 | 40/94 | 12/5 | 6.6 | 2.1–21.2 | 0.001 |
| All | 125/298 | 26/29 | 2.9 | 1.5–5.4 | <0.001 |
| Pan only chewing | | | | | |
| U/3 | | 1/1 | 2.6 | 0.1–64.3 | 0.56 |
| M/3 | | 19/12 | 5.3 | 2.1–13.6 | <0.001 |
| L/3 | | 3/11 | 0.8 | 0.2–3.1 | 0.75 |
| All | | 26/26 | 2.8 | 1.5–5.2 | 0.002 |
| Bidi smoking | | | | | |
| U/3 | 4/16 | 11/8 | 7.1 | 1.1–46.8 | 0.04 |
| M/3 | 14/76 | 60/73 | 6.0 | 2.5–14.5 | <0.001 |
| L/3 | 12/37 | 34/48 | 3.9 | 1.4–10.7 | 0.008 |
| All | 36/139 | 115/144 | 4.0 | 2.3–6.8 | <0.001 |
| Cigarette smoking | | | | | |
| U/3 | | 1/7 | 0.6 | 0.04–8.1 | 0.68 |
| M/3 | | 16/31 | 2.3 | 0.9–6.0 | 0.09 |
| L/3 | | 9/25 | 1.7 | 0.6–5.1 | 0.35 |
| All | | 26/71 | 1.6 | 0.8–2.9 | 0.16 |
| Alcohol drinking | | | | | |
| U/3 | 14/25 | 2/6 | 0.5 | 0.1–3.4 | 0.52 |
| M/3 | 38/127 | 51/53 | 3.4 | 1.6–7.3 | 0.002 |
| L/3 | 35/70 | 20/40 | 0.8 | 0.3–1.6 | 0.47 |
| All | 99/242 | 78/112 | 1.3 | 0.9–2.1 | 0.21 |

**Table V** Adjusted (for alcohol) odds ratios (ORs) among males for different combinations of chewing (Ch) including tobacco (T) and non-tobacco (NT) chewing and smoking (Sm) including bidi (B) and cigarette (C) smoking for entire oesophagus (–, no habit; +, yes habit)

| | *Cases* | *Controls* | *OR* | *95% CI* | P-*value* |
|---|---|---|---|---|---|
| $Ch^- Sm^-$ | 20 | 116 | 1.0 | – | |
| $Ch^- BSm^+$ | 87 | 123 | 5.0 | 2.6–9.5 | <0.001 |
| $Ch^- CSm^+$ | 18 | 59 | 1.9 | 0.9–4.1 | 0.09 |
| $TCh^+ Sm^-$ | 10 | 16 | 4.3 | 1.6–11.6 | 0.003 |
| $TCh^+ BSm^+$ | 13 | 11 | 9.7 | 3.4–27.2 | <0.001 |
| $TCh^+ CSm^+$ | 3 | 2 | 8.6 | 1.2–57.7 | 0.03 |
| $NTCh^+ Sm^-$ | 6 | 7 | 5.3 | 1.5–18.1 | 0.01 |
| $NTC^+ BSm^+$ | 15 | 10 | 11.5 | 4.1–32.4 | <0.001 |
| $NTCh^+ CSm^+$ | 5 | 9 | 4.3 | 1.2–15.1 | 0.02 |

Tables VI and VII give OR estimates for combinations of alcohol drinking and chewing as well as for drinking and smoking respectively. The estimates are also calculated separately for the middle third of the oesophagus. A combination of drinking and non-tobacco chewing and drinking and bidi smoking showed a highly elevated risk particularly for the middle third of the oesophagus.

Drinking was not a significant risk factor below 60 years of age when the entire oesophagus was considered (OR = 1.6; 95% CI 0.9–2.7) but was significant when only the middle third of the oesophagus was considered (OR = 4.0; 95% CI 1.6–10.0). Persons above 60 years of age showed a significant elevation in risk due to drinking for the entire oesophagus (OR = 2.2; 95% CI 1.2–4.1) and a higher elevation for the middle third (OR = 6.1; 95% CI 2.0–18.5).

Of the 343 cases of oesophageal cancer, 236 cases had a microscopically confirmed diagnosis of squamous cell carcinoma. The risks associated with tobacco chewing in both males and females and smoking and alcohol drinking in males were examined separately for this histological type of cancer of the oesophagus. The risks for squamous cell carcinoma were marginally higher among males with tobacco chewing (OR = 2.8 *vs* 2.1) and non-tobacco chewing (OR = 3.2 *vs* 2.4) than when all diagnoses of oesophageal cancers were considered. Other risk factors did not show much difference in the estimates of risk between squamous cell carcinomas and all cancers of the oesophagus, either for the oesophagus as a whole or when the risk was examined separately for each of the segments.

### Discussion

Cancer of the oesophagus is one of the leading sites of cancer in India (National Cancer Registry Programme of India, 1982–1990). The most recent volume of *Cancer Incidence in Five Continents* (Parkin *et al.*, 1992), indicates that women in the Bangalore population cancer registry have the highest AAR of oesophageal cancer but higher incidences elsewhere, have been reported earlier (Doll *et al.*, 1970). There has been a statistically significant increase in oesophageal cancers in Bangalore in both sexes during the past decade (Nandakumar *et al.*, 1991).

Whereas men chew as well as smoke tobacco, women predominantly chew tobacco in this part of India (Anantha *et al.*, 1995). 'Pan' consists of betel leaf, betel nut (areca catechu) and slaked lime which is chewed as such or with tobacco as a major constituent. Earlier reports from this country have demonstrated the association between 'pan' chewing with or without tobacco and 'bidi' smoking and the risk of oesophageal cancer (Jussawalla and Deshpande, 1971; Jussawalla, 1971; Paymaster *et al.*, 1973; Rao *et al.*, 1989). Studies in India of the risk of oesophageal cancer associated with alcohol drinking have also been reported (Notani, 1988; Sankarnarayanan *et al.*, 1991). The present study substantiates these earlier findings and further examines the risks for cancer of each of the three segments of the oesophagus as well as for the different histological types.

Smoking as such, without account of the form (bidi or cigarette), was found to be a risk factor. However, when

**Table VI** Adjusted (for smoking) odds ratios (ORs) among males for different combinations of chewing (Ch) including tobacco (T) and non-tobacco (NT) chewing and alcohol drinking (A) (–, no habit; +, yes habit) for entire and middle third of oesophagus

| | *Ca* | *Co* | *All segments* *OR* | *95% CI* | P-*value* | *Ca* | *Co* | *Middle third* *OR* | *95% CI* | P-*value* |
|---|---|---|---|---|---|---|---|---|---|---|
| $A^- Ch^-$ | 76 | 211 | 1.0 | – | – | 32 | 111 | 1.0 | – | – |
| $A^- TCh^+$ | 13 | 15 | 3.2 | 1.4–7.2 | 0.01 | 2 | 8 | 1.0 | 0.2–4.9 | 0.96 |
| $A^- NTCh^+$ | 10 | 16 | 2.2 | 0.9–5.4 | 0.09 | 5 | 8 | 3.4 | 0.9–12.7 | 0.07 |
| $A^+ Ch^-$ | 49 | 88 | 1.4 | 0.9–2.2 | 0.18 | 29 | 39 | 3.3 | 1.5–7.3 | 0.004 |
| $A^+ TCh^+$ | 13 | 14 | 3.0 | 1.2–7.6 | 0.02 | 8 | 10 | 5.9 | 1.5–22.5 | 0.01 |
| $A^+ NTCh^+$ | 16 | 10 | 5.1 | 2.0–12.6 | <0.001 | 14 | 4 | 29.8 | 6.7–133 | <0.001 |

Ca, cases; Co, controls.

**Table VII** Adjusted (for tobacco and non-tobacco chewing) odds ratios (ORs) among males for different combinations of alcohol drinking (A) and bidi smoking (BSm) (–, no habit; +, yes habit) for entire and middle third of oesophagus

| | All segments | | | | | Middle third | | | | |
|---|---|---|---|---|---|---|---|---|---|---|
| | *Ca* | *Co* | *OR* | *95% CI* | P-*value* | *Ca* | *Co* | *OR* | *95% CI* | P-*value* |
| $A^- BSm^-$ | 48 | 161 | 1.0 | – | – | 18 | 84 | 1.0 | – | – |
| $A^- BSm^+$ | 51 | 81 | 2.8 | 1.7–4.7 | <0.001 | 21 | 43 | 4.5 | 1.9–11.1 | <0.001 |
| $A^+ BSm^-$ | 14 | 49 | 1.0 | 0.5–2.1 | 0.96 | 12 | 23 | 4.4 | 1.7–13.9 | 0.01 |
| $A^+ BSm^+$ | 64 | 63 | 4.6 | 2.6–8.3 | <0.001 | 39 | 30 | 16.2 | 4.7–46.3 | <0.001 |

Ca, cases; Co, controls.

smoking was separated into 'bidi' and cigarette smoking only bidi smoking turned out to be a significant risk factor. Furthermore, bidi smoking was the only factor that showed a significant elevation in risk for each of the three segments as well as for the entire oesophagus. The higher OR for the upper third may be relevant to the fact that bidi smoking is responsible for cancer of the larynx as well as the pharynx and base of the tongue (Tomatis *et al.*, 1990). Since bidis are narrower than cigarettes, the stream of smoke that is inhaled is also narrower and perhaps able to travel into the upper digestive tract as well. This would result in direct contact and perhaps damage to the mucous membranes of the digestive tracts, in contrast to cigarette smoke that mainly diffuses into the alveoli and lungs. This may be an explanation for the higher OR owing to bidi smoking that is observed for the upper third of the oesophagus with the risk diminishing for the middle and lower third. Smokers were predominantly bidi rather than cigarette smokers and this low relative proportion of cigarette smokers could be the reason for the absence of any significant elevation in OR for cigarette smoking.

This study confirms earlier reports (Jayant and Yeole, 1987; Paymaster *et al.*, 1973) that chewing even without tobacco was a significant risk factor in the aetiology of oesophageal cancer. In the present study, risk due to non-tobacco chewing was confined to the middle third of the oesophagus among males. Similarly, tobacco chewing appears to be responsible only for cancer of the lower third segment.

Paymaster *et al.* (1973) examined risks associated with smoking (bidi and cigarette) and chewing (with and without tobacco) for cancers of the different segments of the oesophagus. As in the present study the highest risk was seen for the upper third with a decline in risk for the other two segments. They also found that among males a significant risk due to non-tobacco chewing was seen only for the middle third of the oesophagus. However, unlike this study, the risk estimates due to tobacco chewing were similar to that of bidi smoking, with declining risk for lower segments. It is possible that their risk estimates were not adjusted for smoking and alcohol drinking.

It is difficult to interpret the differences in risk associated with different segments of the oesophagus. A larger sample size, would give a clearer picture. It seems surprising that among females, non-tobacco chewing showed a significant increase in risk for the lower third whereas in males a significant elevated risk was seen only for the middle third. Pan chewing, with or without tobacco, appears to be associated with cancers of the lower third of the oesophagus. The main ingredient of pan when it is chewed without tobacco is areca nut, which possibly interacts with the tobacco of bidi smoke in contributing to risk of cancers of the middle third of the oesophagus.

That alcohol drinking was a significant risk factor for only the middle third of the oesophagus is a new finding of this report. Of additional interest appears to be the increased risk associated with the combination of alcohol drinking and bidi smoking habits on one hand and alcohol drinking and tobacco chewing on the other with substantially higher risk for the middle third segment. Both cases and controls gave a history of consuming mainly the local brands of alcohol. This essentially consists of preparations from extracts of coconut or palm trees and also from molasses. Information on the frequency, quantity consumed and duration of the habit were available, but small numbers did not permit estimation of risk for each segment of the oesophagus.

Cigarette smoking is known to be associated with squamous cell carcinoma rather than adenocarcinoma of the lung. In the present study the risks associated with different combinations of chewing, smoking and alcohol drinking were greater when only squamous cell carcinoma of the oesophagus was considered in the analysis.

The role of diet in the risk of oesophageal cancer in India has been studied (Notani and Jayant, 1987). The dietary patterns practised in India are complex and vary from region to region and within regions. In the present study we were able to gather information on only the two most prominent aspects – vegetarian or non-vegetarian diet and staple cereal. Neither parameter influenced the risk of oesophageal cancer. It is pertinent to note that a 'non-vegetarian diet' does not usually mean that the individual has such a diet on a daily or regular basis: economic conditions would not permit this for most people. It merely means that these persons do consume meat or other animal products on occasions as opposed to vegetarians who never consume them.

Both cases and controls were from the same residential area and the same hospital and were age and sex matched. The failure to adhere to a strictly population-based study with all cases registered for the period being included or the selection of hospital controls (as opposed to population controls) is unlikely to alter the essential findings of the study, since other factors in design and analysis including the random selection of controls from the pool of potential ones would largely offset any such weaknesses in the study. The numbers of cases for the upper third segment were small and for the same reason the risk associated with a combination of different risk factors among males and dose–response parameters could not be adequately assessed.

This study points to the importance of investigating larger numbers of oesophageal cancer cases by subsites and histological types in relation to risk factors.

**Acknowledgements**

The cooperation extended by faculty and staff of Kidwai Memorial Institute of Oncology and that of the participating institutions to the population-based cancer registry is gratefully acknowledged. The authors wish to thank Dr PC Gupta, Senior Research Scientist, Tata Institute of Fundamental Research, Bombay, and Mr P Gangadharan, Emeritus Scientist, Regional Cancer Centre, Thiruvananthapuram, for their valuable comments on the final draft of the manuscript.

### References

ANANTHA N, NANDAKUMAR A, VISHWANATH N, VENKATESH T, PALLAD YG, MANJUNATH P, KUMAR DR, MURTHY SGS, SHIVASHANKARIAH AND DAYANANDA CS. (1995). Efficacy of an anti-tobacco community eduction program in India. *Cancer, Causes Control*, **6**, 119–129.

BRESLOW & DAY, 1980. *Statistical Methods in Cancer Research*, IARC Scientific Publications No. 32. IARC: Lyon.

DOLL R, MUIR C AND WATERHOUSE J. (1970). *Cancer Incidence in Five Continents*, Vol. II. International Union Against Cancer: Geneva.

IARC (1985*a*). *IARC Monographs on the Evaluation of the Carcinogenic Risk of Chemicals to Humans. Tobacco Smoking*, Vol. 38. IARC: Lyon.

IARC (1985*b*) *IARC Monographs on the Evaluation of the Carcinogenic Risk of Chemicals to Humans. Tobacco Habits other than Smoking; Betel-quid and areca-nut chewing; and some related Nitrosamines*, Vol. 37. IARC: Lyon.

IARC (1988). *IARC Monographs on the Evaluation of the Carcinogenic Risk of Chemicals to Humans. Alcohol Drinking*, Vol. 44. IARC: Lyon.

JAYANT K AND YEOLE BB. (1987). Cancer of the upper alimentary and respiratory tracts in Bombay, India; A study of incidence over two decades. *Br. J. Cancer*, **56,** 847-852.

JUSSAWALLA DJ. (1971). Epidemiological assessment of the aetiology of oesophageal cancer in Greater Bombay. In *Monograph on International Seminar on Epidemiology of Oesophageal Cancer*. Jussawalla DJ and Doll R (eds). pp. 20-30, Bangalore.

JUSSAWALLA DJ AND DESHPANDE VA. (1971). Evaluation of cancer risk in tobacco chewers and smokers: An epidemiologic assessment. *Cancer*, **28,** 244-252.

MUIR CS, WATERHOUSE J, MACK T, POWELL J AND WHELAN S. (1987). *Cancer Incidence in Five Continents*. Vol. V. IARC Scientific Publications No 88. IARC: Lyon.

NANDAKUMAR A, ANANTHA N AND THIMMASETTY K. (1991). *Cancer Patterns in Bangalore, India, 1982-1989*. Population-based Cancer Registry of Bangalore, Kidwai Memorial Institute of Oncology: Bangalore.

NANDAKUMAR A, ANANTHA N AND VENUGOPAL TC. (1995). Incidence, mortality and survival in cancer of the cervix in Bangalore, India. *Br. J. Cancer*, **71,** 1348-1352.

NATIONAL CANCER REGISTRY PROGRAMME (NCRP) OF INDIA. (1982-1990). *Annual Reports*. Indian Council of Medical Research: New Delhi.

NOTANI PN. (1988). Role of alcohol in cancers of the upper alimentary tract: use of models in risk assessment. *J. Epiderm. Commun. Health*, **42,** 186-191.

NOTANI PN AND JAYANT K. (1987). Role of diet in upper aerodigestive tract cancers. *Nutr. Cancer*, **10,** 103-113.

PARKIN DM, MUIR CS, WHELAN SL, GAO YT, FERLAY J AND POWELL J. (1992). *Cancer Incidence in Five Continents*. Vol VI. IARC Scientific Publications No 120. IARC: Lyon.

PAYMASTER JC, GANGADHARAN P AND RAO DN. (1973). Some high risk groups in cancer of the oesophagus. In *Proceedings of the Second International Symposium on Cancer Detection and Prevention*. International Congress Series No. 322. pp. 507-519. Bologna: Italy.

RAO DN, SANGHVI LD AND DESAI PB. (1989). Epidemiology of esophageal cancer. *Semin. Surg. Oncol.*, **5,** 351-354.

SANKARNARAYANAN R, DUFFY SW, PADMAKUMARY G, NAIR SM, DAY NE AND PADMANABHAN TK. (1991). Risk factors for cancer of the oesophagus in Kerala, India. *Int. J. Cancer*, **49,** 485-489.

TOMATIS L, AITO A, DAY NE, HESELTINE E, KALDOR J, MILLER AB, PARKIN DM AND RIBOLE E. (1990). *Cancer: Causes, Occurrence and Control*. IARC Scientific Publications No. 100. IARC: Lyon.

TUYNS AJ, PEQUIGNOT G AND JENSEN OM. (1977). Oesophageal cancer in Ille et Villaine in relation to alcohol and tobacco consumption. Multiplicative risks. *Bull. Cancer*. **64,** 45-60.

WATERHOUSE J, MUIR C, SHANMUGARATNAM K AND POWELL J. (1982). *Cancer Incidence in Five Continents*, Vol. IV, IARC Scientific Publications No. 42. IARC: Lyon.